# The Relevance of Assessing Subjective Experiences of Skin Toxicity During Adjuvant Radiotherapy for Breast Cancer

**DOI:** 10.3389/fonc.2021.645921

**Published:** 2021-04-15

**Authors:** Gioia Bottesi, Antonio Stefanelli, Giovanni Ambroso, Gianni Baratto, Eleonora Carraro, Agostino Cristaudo, Laura Giuntoli, Giada Maramaldi, Martino Meneghin, Genny Pozzati, Alessandra Semenzato, Stefano Togni, Giulio Vidotto

**Affiliations:** ^1^ Department of General Psychology, University of Padua, Padua, Italy; ^2^ Radiotherapy Department, Ferrara Hospital, Ferrara, Italy; ^3^ Farmastudio-Fast Srl, Rovigo, Italy; ^4^ Unifarco S.p.A., Santa Giustina, Italy; ^5^ INDENA S.p.A., Milan, Italy; ^6^ Department of Pharmaceutical and Pharmacological Sciences, University of Padua, Padua, Italy

**Keywords:** radiodermatitis, skin toxicity, breast cancer, adjuvant radiotherapy, subjective experiences, supportive care

## Abstract

**Purpose:**

Radiodermatitis is likely to be an inevitable side effect of radiotherapy (RT) but experiencing pain relief during RT might contribute making treatment more acceptable and less impairing. The current study aimed to assess the subjective perceptions and experiences of skin toxicity in a sample of women undergoing adjuvant RT for breast cancer.

**Methods:**

Eighty patients were randomly assigned to one out of two groups: treatment (i.e., a newly developed topical product) and control (i.e., standard-of-care). Patients underwent adjuvant RT for 3 weeks. Clinical assessment of radiodermatitis and self-reported levels of pain, relief, and perceptions of treatment response were collected at the initiation of RT (T1), during RT (T2 and T3), and 2 weeks after treatment completion (T4). To assess changes in skin-related QoL, a subgroup of patients completed the Padua Skin-Related QoL questionnaire at T0 (before the initiation of RT) and at T4.

**Results:**

A comparable timing of onset and severity of radiodermatitis during treatment was observed in both groups. The treatment group reported lower levels of pain and higher levels of relief compared to the control group when skin toxicity was at its highest levels (T2 and T3). Independent of the group, levels of perceived improvements in clinical status increased over time, whereas skin-related QoL worsened from T0 to T4.

**Conclusion:**

Current findings outline the relevance of integrating clinical evaluations of radiodermatitis with patients’ subjective experiences of skin toxicity in interventional studies. Moreover, they provide preliminary evidence about the soothing effect of a newly developed topical product, thus supporting its usefulness of as a supportive care.

## Introduction

Breast cancer is the most common cancer in women (24.5% of all new female cancers worldwide), accounting for 15.5% of cancer-related deaths among women (1). Radiotherapy (RT) is the standard treatment after conservative surgery even in ageing population (2, 3); however, radiodermatitis appears in 74% to 100% of treated patients. In 10% of cases it occurs as severe (Grade 3) radiodermatitis (4). Radiodermatitis can be considered as a side effect of RT as it causes discomfort in patients; it has a negative impact on Quality of Life (QoL) because of changes in body image, clothing selection, and ability to engage in activities of daily living; furthermore, it may reduce compliance to RT (5, 6). Adverse skin reactions include dryness, erythema and, at higher radiation doses, moist desquamation and adnexa destruction; deep ulcerations can also be observed, although only in very severe cases. Furthermore, skin toxicity can result in discomfort, pruritus, and pain (7).

To date, several interventional studies have been conducted in order to explore the effectiveness of topical products designed to manage radiodermatitis, but convincing evidence about their protective effect is still lacking (8–11). Although radiodermatitis is likely to be an inevitable side effect of RT, experiencing pain relief during RT might contribute making treatment more acceptable and less impairing. However, extant literature did not provide systematic information about subjective pain and/or relief across RT (8, 10) or, when assessed, findings supporting the soothing effect of the product under examination were not reported (9, 11–14). This is controversial, given that these products are increasingly used as supportive care to pharmacological treatments for dermatological diseases and they are claimed to improve QoL that, by definition, is a subjective construct (15–17).

Recent qualitative research on this topic indeed has outlined the relevance of assessing the sensations caused by radiodermatitis (e.g., pain) and the beliefs about the prevention/management of radiodermatitis (e.g., the need to make sure that skin slightly improves day by day) in women with breast cancer receiving RT (18). Moreover, findings from a descriptive longitudinal study conducted on 40 women undergoing whole breast 3-dimensional conformal RT showed a significant worsening in skin-related QoL, but not global QoL, across 5 weeks of treatment (19). As a whole, there is evidence suggesting that interventional studies should consider taking into account the patient’s perceptions and experiences of skin toxicity beyond clinical evaluations of radiodermatitis, since they may assume extreme relevance in terms of treatment acceptability and compliance (6, 18–20).

The current study aimed to assess the subjective perceptions and experiences of skin toxicity in a sample of women undergoing adjuvant RT for breast cancer following conservative surgery of breast cancer. Data were collected within a double-blind randomized controlled trial designed to test the effectiveness of a newly developed topical product in reducing the impact of radiodermatitis in such a clinical population. Patients were randomly allocated to one out of two groups: treatment group (i.e., new topical product) and control group (i.e., standard-of-care). The standard-of-care used in the control group was a basic emollient cream characterized by the presence of lipophilic active ingredients (hydrogeneted Polydecene, Butyrospermum parkii butter) and a specific mixture of lipids (ceramide cholesterol and stearic acid) that helps to rebuild the skin’s protective barrier and have a soothing, emollient effect. This cream is free from fragrances and from preservatives that can cause allergies. It is suitable for all dry skin conditions, in particular for wide affected areas in adults and children who have hyper-reactive, intolerant or allergic skin. On the other hand, the newly developed topical product was formulated with the combination of three active ingredients of vegetal origin with soothing and lenitive properties, namely Boswellia Serrata Resin Extract, Zanthoxylum Bungeanum Fruit Extract, and Tamarindus Indica Seed Polysaccharide, which respectively have soothing, anti-itching and moisturizing properties (21–23). The texture of the formula was designed to have a smooth consistence for an optimal compliance and natural excipients, with film forming properties (24). Therefore, compared to the standard-of-care, the new topical product owes its effectiveness to the action of these actives.

Differences between the two products regarding their effect on the onset and the severity of radiodermatitis were assessed. However, the main aim of the current study was exploring group differences in subjective experiences of skin toxicity during RT. Specifically, we assessed: 1) self-reported levels of pain and relief across treatment; 2) perceptions of treatment response (improvements in clinical status) across treatment; 3) changes in skin-related QoL from pre- to post-treatment.

## Materials and Methods

### Participants and Procedure

From 2015 to 2018, 80 consecutive patients at the Radiotherapy Department of Ferrara Hospital (Italy) were enrolled when starting adjuvant RT following conservative surgery of breast cancer. Exclusion criteria were previous or concomitant chemotherapy and a Karnofsky Performance Status (KPS) (25) lower than 60. Informed consent was obtained from all individual participants included in the study. All procedures performed in studies involving human participants were in accordance with the ethical standards of the Ethical Committee of Ferrara Hospital, Italy (approved on date 19/11/2015; approval number: 151092), and with the 1964 Helsinki declaration and its later amendments or comparable ethical standards. When the study was approved, the newly developed topical product was classified as a cosmetic and a clinical trial registration was not required.

Patients were screened for participation in their first visit; eligible participants were then provided detailed information about the study aims and procedure. Patients in both groups were instructed to apply the product they received once a day the week before the beginning of RT, twice daily during the RT, and then once a day in the 2 following weeks. All patients were treated with RT for 3 weeks.

The clinical evaluation of radiodermatitis was performed by a single radiation oncologist at 4 different time points: initiation of RT (first week, T1), during RT (second and third week, T2 and T3 respectively), and T4 (2 weeks post-treatment). Self-reported levels of pain, relief, and perceptions of treatment response across RT were collected the same 4 time points. Moreover, a subsample of 44 patients (treatment group: *N* = 21; control group: *N* = 23) completed a self-report questionnaire assessing skin-related QoL at T0 (before the initiation of RT) and T4. All self-report measures were administered by a single clinical psychologist.

### Adjuvant External Radiotherapy

In keeping with the current practice and according to the criteria of clinical inclusion, patients were treated with conformal RT with linear accelerator and 6MV photons, 3D radiological planning with execution of CT for conformational treatment (PO Pinnacle3 Philips HealthcareBox10.000 5680DA Best The Netherlands) (Philips, Fitchburg, WI, USA). Specifically, each patient underwent a planning CT scan without IV contrast in supine position with both arms above the head. Treatment volume and organs at risk were contoured by the same radiation oncologist on every slice of the planning CTscan. RT treatment planning was performed with Pinnacle TPS, and the treatment was delivered with linac using 6 and 15 mv x-photons and 3d CRT technique (tangent fields). In the original protocol, participants were planned to receive a mean prescription dose of 50 Gy in 25 fractions of 2 Gy/fraction. In July 2016, an amendment to the protocol was presented in order to use a hypofractioned dose of 42.4 Gy in 16 fractions of 2.65 Gy/fraction for 5 days per week. Therefore, 2 patients had to be excluded from the study since they received conventionally fractionated RT before the amendment, whereas all patients finally included in the study received the hypofractionated RT (see Sample Description). The dose was prescribed in adherence to ICRU 50 (International Commission on Radiation Units and Measurements) and the current guidelines. Patients did not receive any additional boost.

### Measures

#### Clinical Evaluation of Radiodermatitis

1. Acute Radiation Morbidity Scoring Criteria for radiation effects **(**Radiation Therapy Oncology Group – RTOG scoring system) (26). Radiation effects are classified as follows: 0 = No morbidity; 1 = Follicular, faint or dull erythema/epilation/dry desquamation/decreased sweating; 2 = Tender to bright erythema, patchy moist desquamation/moderate edema; 3 = Confluent, moist desquamation other than skin folds. Pitting edema; 4 = Ulceration, hemorrhage, necrosis.
2. Common Toxicity Criteria (CTCAE v3. 0) (27). Toxicity is classified as follows: 0 = no morbidity; 1= Faint erythema or dry desquamation; 2 = Moderate to brisk erythema or a patchy moist desquamation confined to skin folds and creases: Moderate edema; 3 = Confluent, moist desquamation >1.5 cm diameter, not confined to skin folds. Pitting edema; 4 = Skin necrosis or ulceration of full thickness; may include bleeding not induced by minor trauma or abrasion; 5 = Death.

#### Self-Report Measures

(1) Levels of pain and relief. Patients evaluated their levels of pain and relief on two separate Visual Analogue Scales (VASs). Each VAS consisted of a 100 mm line. Patients were required to mark the point that better fitted with the level of pain (0, “no pain”; 100, “extreme pain”) or relief (0, “no relief”; 100, “extreme relief”) they were experiencing.
(2) Patient Global Impression of Change (PGIC). This measure was included to assess patients’ perceptions of treatment response (i.e. whether, according to patients, improvement or worsening in clinical status has occurred) with respect to the previous week. Participants were asked to provide their evaluation on a seven-point Likert scale (1, very much worse; 7, very much improved).
(3) Padua Skin-Related QoL questionnaire (PSRQ) (15). The PSRQ is a 50-item questionnaire assessing skin-related QoL on a five-point Likert scale (1, “I disagree at all;” 5, “I agree at all”). It includes four scales: “interpersonal impairment” (mild social problems due to one’s own skin dissatisfaction), “positive feelings and emotions” (positive sensory feelings and emotions in relation to one’s own skin), “negative feelings and emotions” (negative affective states due to skin appearance), and “physical distress and impairments” (physical impairments due to a skin disease). The PSRQ scales demonstrated excellent internal consistency, convergent and divergent validity (15, 28). In a recent study, the PSRQ “interpersonal impairment,” “negative feelings and emotions,” and “physical distress and impairments” scales emerged to adequately discriminate patients with Neurofibromatosis Type 1 (NF1) with major skin involvement and patients with NF1 with minor skin involvement (28).

### Statistical Analyses

The primary endpoint of the study was testing differences between the two products regarding their effect on the timing for the onset and the severity of radiodermatitis. Specifically, the treatment group was expected to show delayed timing for the onset and lower severity of radiodermatitis (as measured by the RTOG scoring system) compared to the control group. *A-priori* power analysis suggested that a sample size of 80 subjects (40 participants in the treatment group and 40 participants in the control group) was required to achieve sufficient power to detect meaningful effects.

To test for differences among groups in the timing for the onset of radiodermatitis, survival analysis methods were adopted. In particular, rates of occurrence of radiodermatitis over time were analysed by the Kaplan-Meier test, and the log-rank test was used to detect differences between the two groups.

Groups differences on both clinical evaluation scores (RTOG, CTC) and patients’ self-report measures (VASs and PGIC) were tested through the conduction of generalized linear mixed models (based on the Poisson distribution). As the main objective was to detect potential differences between RT (T2 and T3) and post-treatment (T4) outcomes, planned contrasts were performed.

Lastly, repeated-measure Analyses of Variance (ANOVAs) 2 (Group) × 2 (Time) were conducted to compare scores obtained on the PSRQ scales by the 2 previously described subgroups across time (T0 vs. T4).

## Results

### Sample Description

Overall, 7 patients dropped out the study (treatment group: *N* = 5; control group: *N* = 2). Among them, 2 patients in the treatment group and 2 patients in the control group withdrew their consent without disclosing the reason; moreover, 1 patient in the treatment group withdrew her consent due to difficulties tolerating the odor of the product. Within the treatment group, 2 patients were excluded due to protocol violation (following the above-mentioned amendment of the protocol).

Clinicians recommended product suspension and steroids administration for 3 patients of the control group due to the occurrence of severe radiodermatitis at different time points (1 at T1 and 2 at T2). Lastly, 3 patients of the treatment group and 1 patient of the control group reported adverse events during RT, leading to product suspension and steroid therapy. Steroid therapy was administered during RT. With specific reference to adverse events, 2 patients of the treatment group showed an early erythematous reaction (at T1 and T2, respectively) and 1 patient showed an urticarial rash at T4; 1 patient of the control group showed an itchy erythema at T3.

### Intention-to-Treat Results

Intention-to-treat analyses were conducted on 73 patients (treatment group: N=35; control group: N=38). **Table 1** shows differences among groups in the main demographic and clinical characteristics.

**Table 1:** Main demographic and clinical characteristics of the treatment and control groups (intention-to-treat analyses).

	Treatment group (*n* = 35)	Control group (*n* = 38)
Age (M ± SD)	60.5 ± 11.7	60.1 ± 11.8
BMI (M ± SD)	24.1 ± 3.9	25.6 ± 4.2
KPS (M ± SD)	99.7 ± 1.7	98.4 ± 5.9
Current alcohol use (“yes”/frequency)	4	10
Current tobacco use (“yes”/frequency)	5	3

BMI, body mass index; KPS, Karnofsky Performance Status.

The survival analysis showed that the two groups did not differ with respect to the timing for the onset of radiodermatitis (*χ^2^*_(1)_ = .30, *p* = .60). In both groups, RTOG and CTC scores were comparable: the main effect of Group and the Group × Time interaction were not significant (all *ps* >.05), whereas a significant main effect of Time emerged (RTOG: *χ^2^_(3)_* = 53.35, *p* <.001; CTC: *χ^2^_(3)_* = 50.41, *p* <.001). In both the treatment and the control groups, the highest skin toxicity values were observed at T3 (*mean* RTOG treatment group =1.14 ± 0.69; *mean* RTOG control group =1.26 ± 0.69).

As far as pain is concerned, no significant main effect of Group emerged (*p* >.05), but the main effect of Time (*χ^2^_(3)_* = 301.58, *p* <.001) and the Group × Time interaction (*χ^2^_(3)_* = 14.43, *p* =.002) were significant. The treatment group reported lower levels of pain than the control group at T2 and T3, whereas at T4 levels of pain were comparable (*z* = −3.61, *p* <.001). With respect to relief, no significant main effect of Group was found (*p* >.05), whereas the main effect of Time (*χ^2^_(3)_* = 349.41, *p* <.001) and the Group × Time interaction (*χ^2^_(3)_* = 15.24, *p* = .0016) were significant. The treatment group reported statistically significantly higher levels of relief than the control group at T2 and T3, whilst at T4 levels of relief were comparable (*z* = 3.88, *p* <.001). Lastly, with respect to PGIC, the main effect of Group and the Group × Time interaction were not significant (all *ps* >.05), but a significant main effect of Time emerged (PGIC: *χ^2^_(3)_*= 92.04, *p* <.001).

### Per-Protocol Results

**Figure 1:**
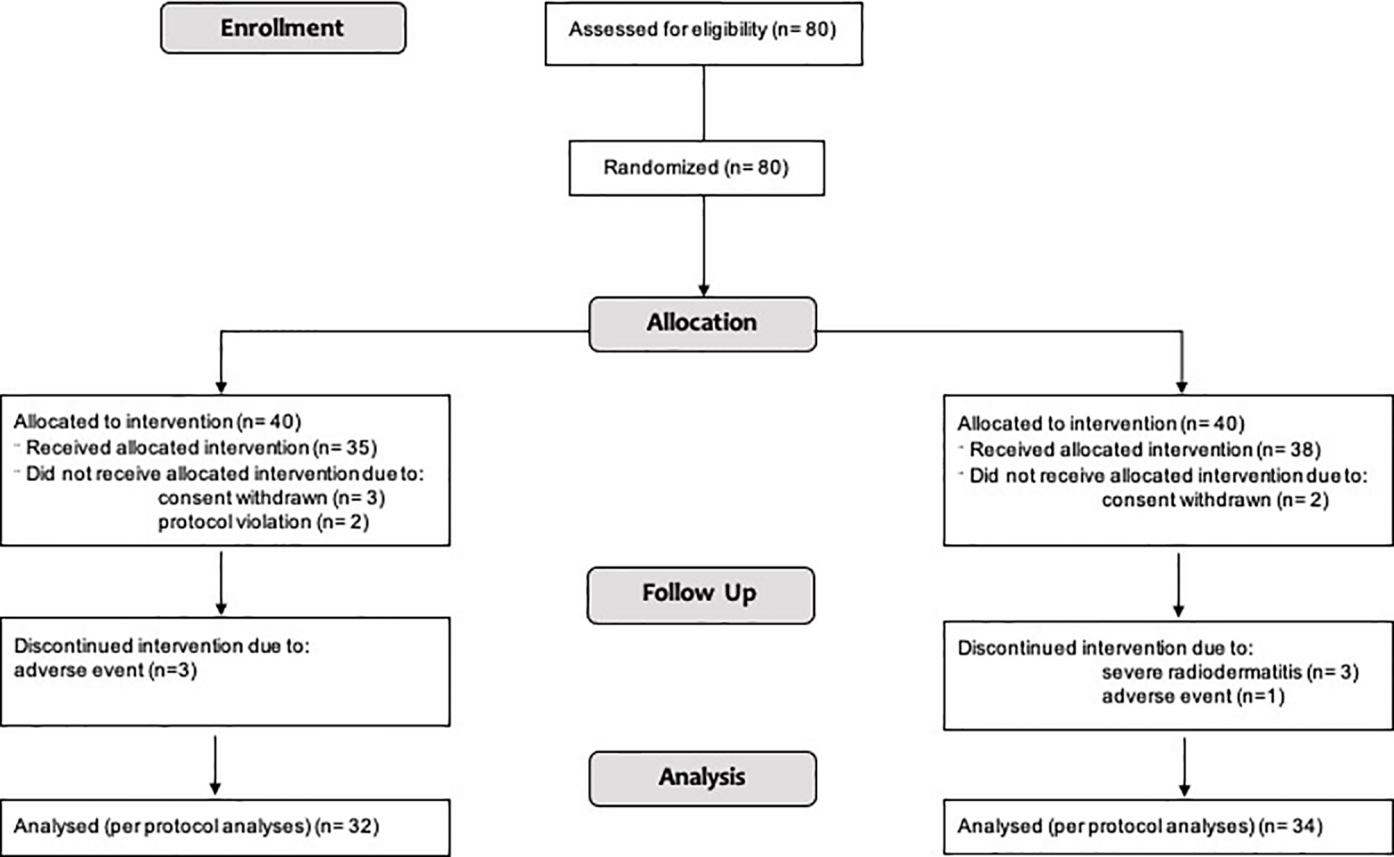
Patient selection (per-protocol analyses) illustrated in a diagram (CONSORT flow chart).

Clinical data from a sample of 66 women (treatment group: *N* =32; control group: *N* =34) were included in the per-protocol analyses (see **Figure 1**). Specifically, patients who discontinued intervention (see Sample Description) were removed from the analyses. 

As shown in **Table 2**, also in this case the two groups were comparable regarding age, Body Mass Index, KPS, alcohol and tobacco consumption (all *p*s >.05).

**Table 2:** Main demographic and clinical characteristics of the treatment and control groups (per-protocol analyses).

	Treatment group (*n* = 32)	Control group (*n* = 34)
Age (M ± SD)	60.5 ± 11.6	61.4 ± 11.4
BMI (M ± SD)	23.8 ± 3.7	25.7 ± 4.1
KPS (M ± SD)	99.7 ± 1.8	98.2 ± 6.3
Current alcohol use (“yes”/frequency)	4	9
Current tobacco use (“yes”/frequency)	5	3

BMI, body mass index; KPS, Karnofsky Performance Status.

#### Group Differences on the Timing for the Onset and the Severity of Radiodermatitis

Findings from the survival analysis showed that the two groups did not differ with respect to the timing for the onset of radiodermatitis (*χ^2^_(1)_* = .00, *p* = .80). Even RTOG and CTC scores were comparable among groups: in both cases, the main effect of Group and the Group × Time interaction were not significant (all *ps* >.05), but a significant main effect of Time was observed (RTOG: *χ^2^_(3)_* = 48.92, *p* <.001; CTC: *χ^2^_(3)_* = 46.99, *p* <.001). In both groups the highest skin toxicity values were observed at T3 (*mean* RTOG treatment group =1.09±0.69; *mean* RTOG control group =1.24±0.65). In particular, as displayed in **Table 3**, during week 3 a mild reaction (RTOG 1–2) was observed in 26/32 of the patients in the treatment group vs. in 30/34 of the control one. A more pronounced reaction (RTOG 3) was seen in 1/34 of the patients in the control group *vs.* in 0/32 of the treatment one.

**Table 3:** Frequencies (percentages) of the RTOG and CTC criteria across treatment.

	Treatment group (*N* = 32)	Control group (*N* = 34)	Treatment group (*N*= 32)	Control group (*N* = 34)	Treatment group (*N* = 32)	Control group (*N* = 34)	Treatment group (*N* = 32)	Control group (*N* = 34)
	T1 n(%)	T1 n(%)	T2 n(%)	T2 n(%)	T3 n(%)	T3 n(%)	T4 n(%)	T4 n(%)
**CTC**								
0	26(39.4%)	27(40.9%)	18(27.3%)	21(31.8%)	6(9.1%)	3(4.5%)	21(31.8%)	23(34.8%)
1	6(9.1%)	3(4.5%)	10(15.2%)	10(15.2%)	19(28.8%)	21(31.8%)	7(10.6%)	4(6.1%)
2			3(4.5%)	3(4.5%)	7(10.6%)	9(13.6%)	4(6.1%)	4(6.1%)
3						1(1.5%)		1(1.5%)
N.A.		4(6.1%)	1(1.5%)					2(3%)
								
**RTOG**								
0	26(39.4%)	27(40.9%)	19(28.8%)	21(31.8%)	6(9.1%)	3(4.5%)	21(31.8%)	24(36.4%)
1	6(9.1%)	3(4.5%)	8(12.1%)	10(15.2%)	17(25.8%)	21(31.8%)	7(10.6%)	3(4.5%)
2			4(6.1%)	3(4.5%)	9(13.6%)	9(13.6%)	3(4.5%)	5(7.6%)
3						1(1.5%)	1(1.5%)	1(1.5%)
N.A.		4(6.1%)	1(1.5%)					1(1.5%)

CTC, Common Toxicity Criteria (CTCAE v3. 0); RTOG, Radiation Therapy Oncology Group scoring system; T1, first week of RT; T2, second week of RT; T3, third week of RT; T4, 2 weeks post-treatment.

#### Group Differences in Self-Reported Levels of Pain and Relief

Mean values (SD) of self-reported levels of pain and relief in the two groups are displayed in **Table 4**.

**Table 4:** Mean values (±SD) obtained by the two groups on patients' self-reported levels of pain and relief (VASs) and on perceived treatment response (PGIC) across treatment.

	**Treatment group (*N*=32)**	**Control group (*N*=34)**
**Pain (VAS)**		
T1	0.91 ± 2.83	0.63 ± 1.96
T2	2.90 ± 5.43	4.85 ± 8.58
T3	6.41 ± 7.36	9.59 ± 10.16
T4	5.00 ± 12.69	5.61 ± 12.27
**Relief (VAS)**		
T1	98.59 ± 4.12	98.84 ± 3.52
T2	95.84 ± 5.63	94.56 ± 8.76
T3	92.66 ± 8.05	89.32 ± 8.86
T4	93.66 ± 16.77	94.09 ± 13.22
**PGIC**		
T1	1.60 ± 1.22	1.41 ± 1.02
T2	1.62 ± 1.07	1.66 ± 1.15
T3	2.57 ± 1.44	2.75 ± 1.38
T4	5.03 ± 2.04	5.03 ± 2.05

VAS, visual analog scale; PGIC, Patient Global Impression of Change; T1, first week of RT; T2, second week of RT; T3, third week of RT; T4, 2 weeks post-treatment.

**Figure 2:**
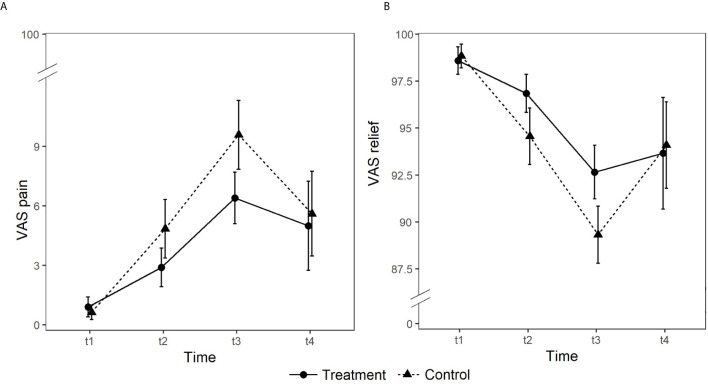
Group differences in self-reported levels of pain (panel **A**) and relief (panel **B**) rated on 0 to 100 visual analog scales (VAS).

With respect to pain, no significant main effect of Group emerged (*p* >.05). On the contrary, the main effect of Time (*χ^2^_(3)_* = 293.22, *p* <.001) and the Group × Time interaction (*χ^2^_(3)_* = 11.90, *p* =.008) were significant. The treatment group reported lower levels of pain than the control group at T2 and T3, whilst at T4 levels of pain were comparable (*z* = −2.13, *p* =.033). Consistently, as regards relief, no significant main effect of Group emerged (*p* >.05). On the contrary, the main effect of Time (*χ^2^_(3)_* = 327.08, *p* <.001) and the Group × Time interaction (*χ^2^_(3)_* = 19.88, *p* <.001) were significant. The treatment group reported statistically significantly higher levels of relief than the control group at T2 and T3, whilst at T4 levels of relief were comparable (*z* = 3.79, *p* <.001) (see **Figure 2**).

#### Group Differences in the Patient Global Impression of Change

As far as the PGIC is concerned, scores were comparable among groups. The main effect of Group and the Group × Time interaction were not significant (all *p*s >.05), but a significant main effect of Time was observed (PGIC: *χ^2^_(3)_* = 92.04, *p* <.001). Independent of the group, levels of perceived improvements in clinical status increased over time. Means (SD) are reported in **Table 4**.

### Group Differences in Skin-Related QoL Over Time

Forty-four patients completed the PSRQ at T0 and at T4 (**Table 5**). The main effect of Group and the Group × Time interaction were not significant (all *p*s >.05) for all the PSRQ scales. A significant main effect of Time was observed for the PSRQ “interpersonal impairment,” “negative feelings and emotions,” and “physical distress and impairment” scales. Specifically, independent from group, patients reported higher scores in these PSRQ scales at T4 when compared to T0 (all *p*s = .02).

**Table 5:** Mean values (±SD) obtained by the two subgroups on the PSRQ at T0 and T4.

**PSRQ scale**	Treatment group (*N* = 21)		Control group (*N* = 23)			
	T0	T4	T0	T4		*p*
Interpersonal impairment	13.67 ± 3.14	15.24 ± 3.66	13.78 ± 3.48	16.65 ± 4.75		0.004
Positive feelings and emotions	46.09 ± 12.05	48.57 ± 11.43	50.65 ± 14.71	55.35 ± 13.74		0.103
Negative feelings and emotions	18.48 ± 7.86	21.95 ± 7.94	17.22 ± 6.99	21.13 ± 6.08		0.008
Physical distress and impairments	10.76 ± 3.13	13.81 ± 4.71	11.04 ± 3.35	12.74 ± 2.83		0.004

PSRQ, Padua Skin-Related QoL questionnaire; T0, before the initiation of RT; T4, 2 weeks post-treatment. p, p-value for the difference between T0 and T4.

## Discussion

Radiodermatitis is one of the main side effects of RT for breast cancer and it can significantly impair both QoL and treatment compliance (5, 6); in this scenario, a well-tolerated supportive care to be applied during RT might represent a substantial relief. Recently, a new topical product formulated with the combination of three active ingredients of vegetal origin has been tested and identified as a promising support in delaying the onset of radiodermatitis-induced effects (24). Since literature recommends the need to include also patients’ subjective evaluations in interventional studies (6, 18–20), in the current study we focused our interest on the perceptions and experiences of skin toxicity of two groups of women receiving adjuvant RT for breast cancer. Specifically, one group (i.e., treatment group) was instructed to use the newly developed topical product, whereas the other one (i.e., control group) a standard-of-care.

Findings from clinical evaluations of radiodermatitis showed that the two products had a comparable impact on the timing for the onset and the severity of radiodermatitis. In both groups, the severity of radiodermatitis showed an increasing trend from T1 (initiation of RT, week 1) to T3 (week 3); a significant reduction in skin toxicity (compared to both T2 and T3) was observed at 2 weeks post-treatment (T4). The highest skin toxicity values were observed at T3 and most of patients reported grade 1 to 2 (mild) toxicity. Literature largely documents that radiodermatitis ranging from grade 1 to grade 2 usually develops during the second to fourth week of RT, independent of the RT schedule^1^ 1Total doses of 50 Gy (on breast) and 60 Gy (on tumor bed) at 2 Gy per fractions delivered in 5 weeks (8); total dose of 5000 cGy given in 200 cGy delivered in 5 weeks (11); stratification by total radiation dose including boost (50.0 cGy to < 59 cGy vs. 59 cGy to 64.o cGy) delivered in 5 weeks (29); midplane dose of 2 Gy per fraction up to a total dose of 70 Gy delivered in 5 weeks (30). and of the use of any product^2^ 2In some studies, a control arm was not included (8, 11, 30). In the case a standard-of-care was delivered, it was Aquaphor, Aloe Vera, or other unspecified therapies (29). (8, 11, 29, 30). Nevertheless, the treatment group self-reported lower levels of pain and higher levels of relief than the control group at T2 and T3. This result suggests that the newly developed topical product was evaluated as more effective than the standard-of-care in reducing pain and increasing relief when skin toxicity was at its highest levels. This is a crucial finding, since pain represents one of the main contributors of poor physical and emotional well-being in cancer patients (31) and the management of RT-induced pain is fundamental for diminishing the likelihood of treatment interruption (32).

Both groups reported perceived improvements in clinical status with respect to the previous week at all time points. Some interpretative biases, including patients’ expectations of improvement when participating in a clinical trial, may positively influence impression of change (33, 34). Also, some memory biases (patients might forget how the situation was precisely the week before) may partially explain this result (33, 34). On the opposite, both groups referred a worsening in specific skin-related QoL areas from pre- to post-treatment; this is reasonable, considering that skin toxicity was still present at T4. Noteworthy, mean scores on the PSRQ over time were within the normative range (15), thus suggesting that impairments in skin-related QoL were detectable but not clinically significant.

The present study is characterized by some limitations. First, the sample size was smaller than required due to dropouts and some issues that led to the exclusion of some patients from the analyses. Furthermore, 2 patients received conventionally fractionated RT before the protocol was amended to hypofractionated RT and they were subsequently excluded from the study; we acknowledge that this may be questionable since they were eligible as per the inclusion criteria. Moreover, only a subgroup of the final sample completed all self-report measures. Therefore, emerged findings need to be interpreted with caution because of possible problems with statistical power. Second, information such as skin type and tumour stage were not collected; we acknowledge that their availability would have allowed providing a more exhaustive description of our sample. Third, among the sensations caused by radiodermatitis we only collected data about pain. Importantly, despite a significant pain reduction was observed in the treatment group compared to the control one, self-reported levels of pain were overall quite low across treatment in both groups. Availing of information about other sensations, such as pruritus, may have allowed obtaining a more complete picture about the impact of the two products on patients’ experiences of skin toxicity.

Our findings appear to corroborate the notion that interventional studies may benefit from collecting data about patients’ subjective experiences of skin toxicity in addition to clinical evaluations of radiodermatitis (6, 18–20). Importantly, gaining this information may inform clinicians about the acceptability and perceived effectiveness of RT. Moreover, it would help health care providers and psycho-oncologists in the design and implementation of educational and psychosocial programs aiming to improve motivation and promote adherence to treatment in this specific clinical population. Lastly, in the case at hand, findings from self-report measures allowed concluding that the newly developed topical might represent a promising supportive given its ability to reduce perceived pain when skin toxicity was at its highest levels. To note, it would have been impossible drawing such a conclusion exclusively relying on findings from clinical evaluations of radiodermatitis.

Current findings emphasize the utility of integrating clinical evaluations of radiodermatitis with patients’ subjective experiences of skin toxicity. Thus, a straightforward assessment of patients’ perceptions and skin-related QoL in both interventional studies and clinical practice is highly encouraged. Moreover, the present study provided preliminary evidence about the soothing effect of a newly developed topical product, thus supporting its use to mitigate radiodermatitis-induced skin related side effects in women receiving RT for breast cancer.

## Data Availability Statement

The datasets used and analysed during the current study are available from the corresponding author on reasonable request.

## Ethics Statement

The studies involving human participants were reviewed and approved by the ethical committee of Ferrara Hospital, Italy. The patients/participants provided their written informed consent to participate in this study.

## Author Contributions

GBo, ASt, and GV made substantial contributions to the conception of the work. GBo, GA, GBa, GM, MM, GP, ASe, ST, and GV contributed to the design of the work. EC and LG had a substantial role in the acquisition and analysis of data. GB, AC, and GV had a substantial role in the interpretation of data. GBo, AC, LG, and GV have drafted the work and substantively revised it. All authors contributed to the article and approved the submitted version.

## Funding

The authors declare that this study received funding from Unifarco S.p.A. and INDENA S.p.A. The funder had the following involvement with the study: generation of the product tested in the study, approval of study design and approval of manuscript for publication.

## Conflict of Interest

GA is the president of Farmastudio-Fast Srl and GP was employee at Farmastudio-Fast Srl. At the time of the study, GM, MM, and ST were employees at INDENA S.p.A. GBa is the Scientific Director of Unifarco S.p.A.

The remaining authors declare that the research was conducted in the absence of any commercial or financial relationships that could be construed as a potential conflict of interest.

The authors received funding from Unifarco S.p.A. and INDENA S.p.A. The funders had the following involvement with the study: generation of the product tested in the study, approval of study design and approval of manuscript for publication.
